# Progress of Syphilology

**Published:** 1874-09

**Authors:** James Nevins Hyde

**Affiliations:** Lecturer on Dermatology and Syphilis, Rush Medical College, Chicago


### Progress in Medical Sciences.

ARTICLE I.—*Progress of Syphilology.* By JAMES NEVINS HYDE, M.D., Lecturer on Dermatology and Syphilis, Rush Medical College, Chicago.

1. Triple Ocular Paralysis of Syphilitic Origin. ALFRED FOURNIER. (*Annales de Dermatologie et de Syphiligraphie*, T. 5, No. 3.)

2. Parasitic Balano-Prosthitis in Diabetes Mellitus. DE BEAUVAIS. (*La Gazette Medicale de Paris*, No. 18.)

3. On Tertiary Syphilis. ALFRED FOURNIER. (*La France Medicale*, Nos. 41 and 44.

4. The Effect of Menstruation upon Cutaneous Disorders. DANLAS. (*La Gazette des Hopitaux*, No. 55.)

5. Osseous Lesions in Infantile Syphilis. PONCET. (*Le Progres Medical*, No. 17.)

6. The Archives of Dermatology. L. DUNCAN BULKLEY, M.D.

1. Marie S——, twenty-two years old, of lymphatic temperament, presented herself at the Lourcine Hospital, in June, 1873. She possessed naturally a strong constitution, and had enjoyed previous good health.

She was found to be affected with divers ocular paralyses, about to be described, which had produced a facial expression of remarkable character.

She stated, in response to questions regarding her antecedent history, that she had contracted syphilis four years prior to the date of her examination. At that time she had been admitted to the Lourcine Hospital for various syphilitic lesions, and notably for "a roseola which covered the surface of the body, and mucous patches of the vulva." Some months later, an extensive alopecia occurred. She had, further, been affected at different times with severe attacks of sore throat and ulcerations of the buccal cavity, requiring several cauterizations. Finally, three years ago, she was prematurely delivered of a dead fœtus. Specific treatment had never been pursued, neither in nor out of the hospital. She affirmed that she had never taken either mercury or the iodide of potassium. The only remedies which she acknowledged having employed were "tonic powders."

She believed that she was relieved of the disease, since for two or three previous years no indications of it had existed, when, four months ago, she was seized with severe attacks of headache, coming on at night, and especially during the latter half of the night. Toward four o'clock in the morning, these pains became intolerable, and precluded the possibility of sleep. For about one month they continued with unabated intensity, but diminished, later, without the intervention of treatment.

From this date the patient began to be conscious of another phenomenon, slight at first, but soon aggravated. Occasionally she was annoyed by double vision, which resulted finally in habitual diplopia. To gain an exact idea of the number and situation of objects, she was under the necessity of closing the left eye with the hand, and of using the right eye only for purposes of vision.

In a short time the upper lid of the right side began to droop, and this prolapse increased until the globe was permanently half concealed by the lid.

The disorder of the eyes increased finally to that point, when the patient was compelled to seek medical advice. She consulted M. Desmarres, who recognized the syphilitic character of the paralysis, and ordered some mercurial pills, and a solution of the iodide of potassium. This treatment she discontinued after perseverance in it for a few days, since the headache disappeared, and she thought that a spontaneous cure of the ocular disease would follow. In this she was mistaken, and hence determined to return to the Lourcine.

We first saw the patient in June of 1873. At that time the following conditions were established:

(1.) On the right side, *evident paralysis of the third pair.* This paralysis was declared by its normal symptoms, viz.: (*a.*) Drooping of the upper lid; this drooping, partial, since the eye was not completely, but only about two-thirds, covered; and complete abolition of the power of voluntary elevation of the lid. When the patient desired to employ the right eye in vision, it was necessary for her to raise its upper lid by the finger. When the lid was raised by the hand of another, no spasmodic resistance was encountered, and the removal of the finger was followed by the passive return of the lid to its former situation. (*b.*) Fixation of the ocular globe; cornea and pupil in median line; and almost complete loss of power to turn the eye upward, downward, or inward. (*c.*) Marked dilatation of the pupil. Vision was, however, unimpaired. There was no injection of the eye, nor cloudiness of the transparent media.

(2.) On the same side (the right) there was paralysis of the sixth pair; absolute abolition of all power to turn the ocular globe outward.

(3.) On the left side the phenomena were different. Here there was neither ptosis nor pupillary dilatation, and the visual power was unimpaired. The eye perfectly executed all normal movements upward, downward and inward; but there was slight convergent strabismus. The power to turn the eye outward was, however, completely lost. There was then, on this side, paralysis of the sixth pair.

Her general health was fair; her skin free from eruption; there were no lesions of the vulva nor of other mucous surfaces; no adenopathy existed. The headache had been relieved, but the patient was occasionally subject to vertigo, migraine, and dizziness, attributed to the weakness induced by the ocular disease. There were some wandering pains in the right arm and shoulder of the same side.

The following treatment was ordered: Daily frictions, with one drachm of strong mercurial ointment; gargles of the chlorate of potash, to preclude or moderate ptyalism; three teaspoonsful of syrup of iodide of potassium (gr. iv ad. dr. j), and sulphur baths.*

* The mode of administering mercury, here recommended by Dr. Fournier, is worthy of note. Mr. Jonathan Hutchinson, in a paper read before the Hunterian Society, on the 8th of January, takes occasion to observe, that "the inunction method is safe and very efficient, but it is dirty and inconvenient, and probably nothing can be said in its favor which may not be alleged with greater force of the vapor bath." He adds, however, that Drs. Diemer, Wetzlar, Brandis, and Ziemssen, of Aix-la-Chapelle, believe inunction to be the preferable method.

Whether it be due to the fact that syphilis manifests itself largely upon the integumentary structures, or to the circumstance that the disease gains access to the system by a superficial lesion, certain it is that treatment by external applications early recommended itself to the profession. Subsequently little importance was assigned to the subject, from the circumstance that eminent syphilographers neglected to put forward its real value. But it is clear that there is a distinct reaction in the minds of medical men in favor of a return to treatment by the skin, and especially to the inunction of mercury. Prof. Lewins' hypodermic injections of corrosive sublimate, now extensively employed upon the Continent, and the increasing use of the oleates of mercury externally, indicate the direction of this current of opinion. I think it proper to take exception to the remarks quoted above from Mr. Hutchinson. A friend recently informed me that the patients in the venereal wards of the Vienna Hospitals were daily ranged into a long line, and each individual assiduously engaged himself in rubbing mercurial ointment into the loins of that one in the line immediately in front of him, under the direction of the hospital staff. Fournier himself deserves the credit of having, by his recent writings, called the attention of the profession to the real merits of this mode of treatment.

As to the comparison instituted between inunction and vaporization, it may be remarked, in favor of the former: 1st, that it can be accomplished by the individual unassisted by attendants; 2d, that it dispenses with elaborate and expensive apparatus: 3rd, that, when properly performed, the metal is made to penetrate the orifices of the emunctories of the skin—probably the sole avenue of ingress—and thus gains access to the sub-epidermic tissues; while it is admitted that the vapor merely deposits an exceedingly delicate stratum upon the superficies of the skin, with the exception of the rare instances where it is allowed to enter the respiratory passages; 4th, that the baths advised by Fournier do away with the objections on the ground of cleanliness, though it is in many instances, without

June 17. The treatment has been rigorously pursued. There has been some amelioration of symptoms, notably of the paralysis of the third pair on the right side. The treatment was continued, the amount of ointment used at each inunction being increased to two and one-half drachms.

June 24. Great improvement. The left eye could be turned outward almost to the external commissure. On the right, the lateral movements of the ocular globe were almost normal, from one commissure to the other, and the downward movements were in a similar degree restored. The eye could not, however, be turned completely upward, though this could be effected to a greater extent than was possible during the last few days. The pupil was still dilated, but the ptosis was so far diminished that at least one-half of the ocular globe could be unveiled. The diplopia, however, persisted. The treatment was continued, and the syrup progressively increased to four, five and six teaspoonsful daily.

July 1. The improvement continued, but was less noticeable than at the last date. The patient had discontinued the inunction because she had heard that "mercury was injurious to the teeth." Her mouth, gums and teeth were nevertheless free from any mercurial effects, and the treatment was consequently resumed.

July 8. The outward movement of the left eye was comparatively restored. Three-fourths of the right were exposed beneath the lid, and movements in every direction were executed with great facility, though the pupil remained dilated.† The general condition of the patient was at this time exceedingly satisfactory. Since the inauguration of the treatment she had gained considerably in flesh, a circumstance which she herself was the first to note, and which she said had also been remarked by her acquaintances. Her appetite, stimulated possibly by the iodide, was excellent. No stomatitis had occurred. Treatment: Six teaspoonsful of the syrup, inunction with four drachms of mercurial ointment, gargle of chlorate of potash, and sulphur baths.

doubt, preferable to order that the underclothing of flannel should be worn unchanged night and day during the persistence of treatment.

† The order displayed in this restoration of function suggests the following observation: A patient recently consulted the writer, whose first symptom of constitutional syphilis was the early form of iritis. Prior to infection there had been aggravated ophthalmia of the same eye, resulting from rubeola. This would seem to explain the selection of that organ for the evolution of the first one of the so-called secondary symptoms. The question arose: Would impairment of function in the nerves supplying any portion of the body determine to that part the manifestations of syphilis? Curiously enough, the question was soon answered by a male patient, who presented himself with long-standing paralysis and marked emaciation of the left lower extremity—sensation being unimpaired. He had recently contracted syphilis, and exhibited blotches over the surface of the body, the left leg alone remaining free from indications of the disease.

July 15. The treatment had been regularly pursued. The lid of the right eye was completely replaced; both eyes accomplished all normal movements, and the patient considered herself cured. The pupil, however, remained slightly dilated, and an ephemeral diplopia was still developed by certain movements of the eye-ball.

From this time we lost sight of the patient, till January 21, 1874, when she was induced to return, in order to consult us respecting a new ailment. She then exhibited a gummy tumor of the right leg, of four months' duration, which had not been submitted to treatment.

At this date the contents of the gummy tumor had been evacuated, and there resulted an excavated ulcer, as large as a silver quarter of a dollar, with clean-cut edges, a yellowish base, presenting still some traces of granulations where degeneration of the syphiloma had occurred, and a deep brown areola. The patient declared that the disorder of the eyes was completely relieved, and there was no evidence to contradict this statement. It was clear, on examination, that the once paralyzed muscles could execute their physiological movements. The pupil was no longer dilated, and the diplopia had disappeared.‡

The recurrence of syphilitic manifestations after an energetic treatment pursued for several weeks, is not surprising, since the disease develops itself in this manner with great frequency. Its career is but temporarily checked by the intervention of specific remedies, and, after brief pauses, is apt to display renewed activity. In order to gain a complete mastery over it, not only in the present, but in the future—in order to completely extinguish the diathesis—it is necessary, as I have elsewhere remarked (see No. 3 of this *Report of Progress*), to resort to multiplied and repeated courses of treatment. "Long, indeed, should be that antisyphilitic medication, from which are to be obtained not only those results which are evident, but those which directly influence the diathesis. A chronic disorder requires a chronic treatment—this is the rule. A syphilitic dyscrasia is neither modified, corrected nor annulled by any other than a long medication—a pro-

‡ Dr. W. H. Broadbent, in the first of his valuable lectures upon Syphilitic Affections of the Nervous System, recently published in the *Lancet*, remarks as follows:

"The twofold fact, that the third nerve is the most frequently affected and often the only one paralyzed, and, again, that portions of the nerve may suffer before the others, is explained by the habit of syphilitic exudation, of which the interpeduncular space at the base of the brain, traversed by the third nerve on its way to the cavernous sinus, is the favorite seat; and it is conclusive evidence that the paralysis is not due to periostitis at the orifice of exit. The nerve also has been found compressed by a gummatous tumor of the sella turcica of the sphenoid bone. * * * * * Paralysis of the sixth, the evidence of which is internal strabismus, is, perhaps, next in frequency."

cess of depuration, greatly extended and oft-repeated—in brief, by a chronic treatment." In the present case, the production of the syphiloma, consecutive to that of the ocular lesions described, is a fact in perfect accordance with our usual and daily experience. It simply testifies to the existing intensity of the disease, which it is important to control by a prolonged and energetic treatment.

But the interest attaching to the case reported does not lie solely in this fact. It is to be found in the *multiplicity of the paralytic symptoms* presented by the patient. Three nerves were involved—the third and sixth pairs of the right side, and the sixth pair of the left. That these three nerves were affected by syphilis, is evident from the mode of evolution of the disease, and the success of the specific remedies employed.

Now this *association of several forms of paralysis* is worthy of note. It is of more frequent occurrence in syphilis than in any other disease, as I have had occasion to demonstrate in numerous instances. It has, then, in a diagnostic point of view, a special significance. It is to this fact that the details given above testify, and I have here limited myself to the mere statement of the case, reserving for a special paper the considerations which it seems to me to suggest.

2. Gubler was the first to establish the fact, that saccharine urine, lodged in the præputial sac, undergoes lactic or acetic fermentation, in consequence of the presence of mucedinous spores in the secretions of the mucous membranes. Genuine balano-prosthitis is apt to result, accompanied by itching and tingling sensations, and sometimes producing a phymosis, for which the patient consults his physician. It is at this point that M. de Beauvais finds a fact of great clinical value.

If the causative relation of these two disorders be ignored, and no suspicion of the urinary disease exists in the mind of the practitioner, after trying in vain a purely medical treatment, he may advise surgical interference. Now this operation, like many others, is liable to serious complications in the case of diabetic patients, and the author cites cases of gangrene of the penis and scrotum, resulting from surgical procedures under these conditions.

It is evidently of great importance to determine the nature of the balano-prosthitis. Microscopic examination of the pus or muco-pus found in the præputial cul-de-sac, will, of course, disclose the presence or absence of the mucedinous spores, which are the sole causes of the disease. In the first event, diabetes mellitus should be sought for, and the urine analyzed. If sugar be discovered, all operative interference should be discouraged for the relief of phymosis, and topical treatment of the balano-prosthitis be conjoined with that general treatment appropriate to this constitutional disease.

It should be added that the species of irritation of the sexual organs, described above, is not peculiar to the male sex, but is found also in diabetic females. The same process of fermentation, discovered in the præputial cul-de-sac, here occurs in the vestibule of the vulva, and, extending to contiguous parts, produces an irresistible pruritus. The muco-cutaneous site of the lesion, and its illy-defined borders, taken in connection with some thickening and moderate desquamation of the derma, render the diagnosis clear. Here, also, the analysis of the urine and the microscopy of the vulvar secretion are essential to the recognition of the cause.

3. Tertiary syphilis comprises that group of syphilitic manifestations which occur after long periods succeeding contamination. The disease, in its tertiary periods, tends to a spontaneous disappearance; while, in some subjects, it brings about a fatal termination. The reason for this difference in its issues is to be found either in the disease itself, or in the character of the soil in which it is planted, or in the insufficiency of treatment.

(*a.*) To say that we find benign, virulent, grave and inveterate forms of syphilis, is to offer no explanation founded upon the nature of the disease. It is simply stating a fact. It is often true that the disease which is benign in its early periods, is brought to a conclusion in the tertiary stage.

(*b.*) The conditions pertaining to the individual who is the subject of syphilis, are important. These may be: Native or acquired constitutional feebleness, lymphatic temperament, anæmia, scrofula, old age, alcoholism, and the general causes of depression. Yet, however real these influences may be, they are not necessarily effective. Not unfrequently we encounter individuals affected with tertiary syphilis, whose conditions are exactly opposed to those described.

(*c.*) The true reason for the extension of syphilis to the tertiary stage, is the absence or insufficiency of treatment. Neglected syphilis, whatever may be said by authors to the contrary, is, in the majority of cases, certain to assume tertiary phases; and daily experience teaches us that expectant treatment, when employed in the disease under discussion, is sure to be followed by disastrous results.

*Chronology.* Three points of interest are here to be noted: the mode of transition from secondary to tertiary types; the epoch of the appearance of the first manifestations of tertiary syphilis; and the duration of the latter period.

As to the first, it is, of course, impossible to establish a fixed mathematical limit between the two stages. This is proven by the fact that such lesions as pustulo-crustaceous syphilides, rupia, ecthyma and sarcocele, can be indifferently ranged in either group. It is to these transitional forms that such names as secondo-tertiary, transitionary, etc., have been applied.

Secondary symptoms are transformed into those of a tertiary type in one or the other of the following methods. First: Contemporaneous incidence of the two periods. In these cases a patient presents simultaneously a cutaneous syphilide and a syphiloma — this is common, but not in accordance with the rule. Or, second, (and much more frequently) there is a more or less complete and more or less prolonged cessation of syphilitic phenomena between the two stages of the disease.

The date of the onset of tertiary forms is very variable. Numerous conditions can defer this explosion, and among them none is more effective than prolonged and energetic treatment. Oftener, tertiary syphilis is not developed before the second or third year, and still more often before the third.

As to the limits of the tertiary period, we know that they may be very much restricted and long deferred. Ten, twenty, thirty years have elapsed after the appearance of the primary lesion, before tertiary syphilis has been developed, and in some rare instances forty, forty-two and forty-five. In one instance, M. Fournier treated a patient seventy-two years old, who had caries of the inferior maxilla and a gummy tumor of the thigh, respectively fifty-two and fifty-five years after the existence of chancre.

*General characters of the tertiary stage.* In general, we discover a condition of apparent health, interrupted, at indeterminate intervals, by various morbid explosions of a special character. Beyond certain exceptional cases, the man affected with tertiary syphilis is in fair health, but under the influence of a morbid yet dormant ferment, whose dangerous portents are often as inappreciable by himself as by his physician.

Secondary accidents have the following distinct features: — a determinate onset, preceded a few weeks by chancre; a group of pathological symptoms, succeeding each other at comparatively short intervals, and limited to a certain period; and a settled termination at the end of two or three years, after which these phenomena are not established.

Tertiary accidents are quite different. They may appear after two or forty years. When numerous, they are not associated, and may be separated by long intervals. They are unlimited as to time; it is impossible to fix a date beyond which they shall not be declared. More precisely, they have the four following distinguishing features:

(*a.*) Tertiary manifestations are discrete, isolated and solitary.

(*b.*) These manifestations are insidious in their approach, slow of development, and often latent as regards their evolution.

(*c.*) They involve the parenchyma of tissues. While secondary accidents are superficial and injure the tissues but slightly, those of a tertiary type have a profound effect. Ulceration, disorganization, mutilation and atrophy follow in their track. The prognosis of the one form is favorable; of the other, serious. In the latter case some of the symptoms are common in non-specific disorders. Syphilitic laryngeal phthisis, for example, does not differ greatly from that of tuberculous origin — the gummy tumor produces a paralysis not different from any other, when it impinges upon a nerve—vomicæ resulting from ulcerated syphilomata resemble ordinary pulmonary excavations. Syphilis in its old age, loses the imprint of a venereal affection;—according to the phrase of Ricord, it "assumes a virtuous expression."

(*d.*) Tertiary manifestations are, with certain rare exceptions, amenable to treatment.

Syphilis invades the entire domain of the body in its tertiary forms; and in spite of the multiform character of the lesions of the latter, we can recognize in them but two distinct pathological processes—the sclerotic and the gummy.

As regards diagnosis, we must look first to the peculiar character of the lesion; second, to the coexistence of specific phenomena; and, third, to specific antecedents. But, per contra, there are few lesions of tertiary syphilis which exhibit a pathognomonic symptomatology, and in general these lesions are isolated and unaccompanied by other indications of disease which would make the diagnosis facile and sure. In addition to all this, syphilitic antecedents, especially in women, are often forgotten, misunderstood or concealed; while the long interval which is apt to occur between early and late manifestations, constitutes a source of error. The situation, moral character and social positions of patients, is not without influence upon the mind of a practitioner.

*Prognosis and Treatment.* The two are intimately associated—the former, always serious, often grave, and, more often than is generally believed, fatal. Due weight should be given to the seat, extent and stage of the disease, as well as to the condition of the patient. "Pox once recognized, is half cured," is a common saying, and never more true than in tertiary syphilis, which is more amenable to specific treatment than either primary or secondary disease.

The iodide of potassium is marvellous, incomparable, in its effects; but even in combination with mercury, is not infallible. If the intervention of the remedy be too late, or if, as in cerebral syphilis, morbid processes of non-specific character are induced by the syphiloma, rebellious syphilis results. Some cases are altogether beyond the reach of therapeutics — they are always in curing and never cured. A fatal evolution of the disorder occurs, and lesion after lesion is produced in spite of all the remedies employed.

Syphilis, is, in general, not as formidable in our eyes as it is in reality. Every syphilitic is not curable, as many suppose, because he is syphilitic. They who are most familiar with the scourge, entertain the greatest dread of it. If then, in conclusion, syphilis exposes its victims to the gravest danger in the distant future — a danger whose consequences we are but too often powerless to avert — it is of the greatest consequence that it be combatted *ab ovo*.

Prevention is of much more ready accomplishment than cure. All treatment therefore should be instituted at the debut of the diathesis. In treating a case of incipient or recent syphilis, the result to be attained by the physician is the elimination of the possibility of the future occurrence of tertiary disease. The ideal to be kept in view is the extinguishing of the diathesis, as a safeguard for the future; and, to secure this end, immediate, energetic and prolonged treatment is requisite.

4. There is a manifest sympathy between the uterus, ovaries and integumentary system. This sympathy declares itself either in the form of eruptions whose appearance coincides with menstrual epochs, or in the development of uterine derangements, or in the disappearance of morbid cutaneous phenomena at the arrival of puberty, or after the relief of dysmenorrhœa. In explanation of this, it may be said that the affections of the skin present a striking analogy to diseases of the nervous system, —some of them exhibiting a hyperæsthesia (herpes) — some, a neuralgic condition (zona) — some others an anæsthesia (lepra); while it may be remarked that of all the systems of the human economy, the nervous is that which is the first to reflect disorders of the womb (hysteria.) We are thus enabled to appreciate the fact that cutaneous diseases connected with menstruation or any of its derangements, are in fact reflex neuroses. It should be added, that this sympathy is not evident in the case of all patients, and that in comparatively few, is it established in a high degree.

5. Poncet reports to the Anatomical Society the result of his observations of the bodies of newly born infants, affected with syphilis. Of the twelve cases reported, some presented evident external symptoms of the disease, and the others in every instance were born of parents—father or mother—equally affected. It would appear from these examinations that alteration of the osseous tissue in newly born syphilitic children, is the most frequent of all the symptoms of infantile syphilis, occupying, in fact, the same rank with the mucous patch of the adult.

It should be added, by way of parenthesis, that this observer has concluded that the changes noticeable in the dental enamel, are to be attributed to the maceration of the fœtus in the amniotic liquid; since he has not only discovered this condition in the bodies of non-syphilitic infants, but has also produced the same, artificially, by macerating the maxillæ and teeth of dead-born infants for several days in the same fluid.

We have space merely for the following table, which summarizes the observations referred to :

| No. of Case. | Parental Syphilis. | History of Parents. | Age of Child. | External Symptoms. | Lesions of Osseous Tissue. |
|---|---|---|---|---|---|
| 1 | Unknown. | Unknown. | 3 wks. | Mucous patches of anus and labial commissures. | Slightly marked alteration of bones. |
| 2 | Unknown. | Unknown. | 1 mo. | Coryza. | Pus in right elbow and both hip joints, from arthritis of soft parts. Articular surfaces sound. Central alterations of all osseous tissue. |
| 3 | Abortion at 6¼ months. | Two previous abortions at three and four mos. No evident syphilis. | Dead born. | | Special lesions of bones. |
| 4 | No signs of syphilis in mother. | Mother had been mistress of several syphilitic patients. | 3 mos. | Eczematous eruption. | Bones easily fractured. Slightly marked rachitis |
| 5 | Mother: mucous patches of throat & vulva | Untreated secondary syphilis appeared 3 months before. | Dead born. | | Characteristic alteration |
| 6 | Mother : after confinement, no appearance of syphilis. | In hospital ten years before for mucous patches ; 17 months before, same hospital, syphilitic treatment. | 3 d'ys | | Sound tissue. |
| 7 | Mucous patches of vulva. | Secondary syphilis, untreated ; 2 mos. after conception. | Dead born. | | Special lesions. |
| 8 | Mother, non-syphilitic. | Father, aged 21, had chancre at 15 years. No treatment. | Only 3 or 4 respirations | Large bullæ of pemphigus. Papules on dorsum and venter. | Epiphyses of long bones all mobile, separated and held only by periosteum. Pus beneath the latter in left femur; radius and humerus of both superior extremities. |
| 9 | Abortion at 6½ months. | Secondary syphilis appeared one month after conception. | Dead born. | | All epiphyses of long bones separable and moderately mobile. |
| 10 | Mucous patches of throat. | 7 months pregnancy. | 3 d'ys | | Sound tissue. |
| 11 | Non-syphilitic mother. | | 12 d's. | Papular eruption. | Special lesions. |
| 12 | Mother treated in hospital for secondary syphilis. | | 6 mos. | | Evident rachitis. |

6. On the 1st of October next, it is expected that the *Archives of Dermatology* will appear, from the press of G. P. Putnam's Sons, under the editorial management of Dr. L. Duncan Bulkley. This Journal will be issued quarterly, under the auspices of the New York Dermatological Society, and will be devoted to Diseases of the Skin and allied affections. It is designed to represent particularly the American School of Dermatology, while an epitome of the current literature of this subject in our own and foreign countries, will be published in each number.

The pages will be arranged under the following heads: 1, Original Articles; 2, Transactions of the New York Dermatological Society, including papers, cases and discussions; 3, Clinical Reports; 4, Extracts and Translations from other Journals; 5, Digest of Dermatological Literature; 6, Reviews and Book Notices; 7, Correspondence, and Miscellaneous Matter. Suitable illustrations will be inserted as required, and special attention will be given to photography and microscopy.

We take pleasure in making an announcement of this character, and are desirous of giving a welcome in advance to the project of our co-laborers. They enlist our sympathies in the mere announcement of their design. We have looked for the work of our American Dermatologists in those pages of the *American Journal of Syphilography and Dermatology* which are consecrated to original communications, and we have looked in vain. We find there, translations of foreign articles, which are already in our hands in the journals which first produced them—translations which, while they are often defective and poorly executed, yet, in the main, possess an excellence which renders them valuable to the general student of this species of literature.

We look to the *Archives of Dermatology* to give expression to the opinions and work of the American School of authors—of whom we have every reason to be proud—and wish it may meet with that success which it will be agreed that it deserves.

